# Supplementary material for: Complex‐centric proteome profiling by SEC‐SWATH‐MS
Source: Mol Syst Biol. 2019 Jan 14;15(1):e8438. doi: 10.15252/msb.20188438 (PMC6346213; doi:10.15252/msb.20188438)
Supplement: Supplementary file 8 — Dataset EV7 [file MSB-15-e8438-s008.zip › feature_plots_string/P17812.pdf]

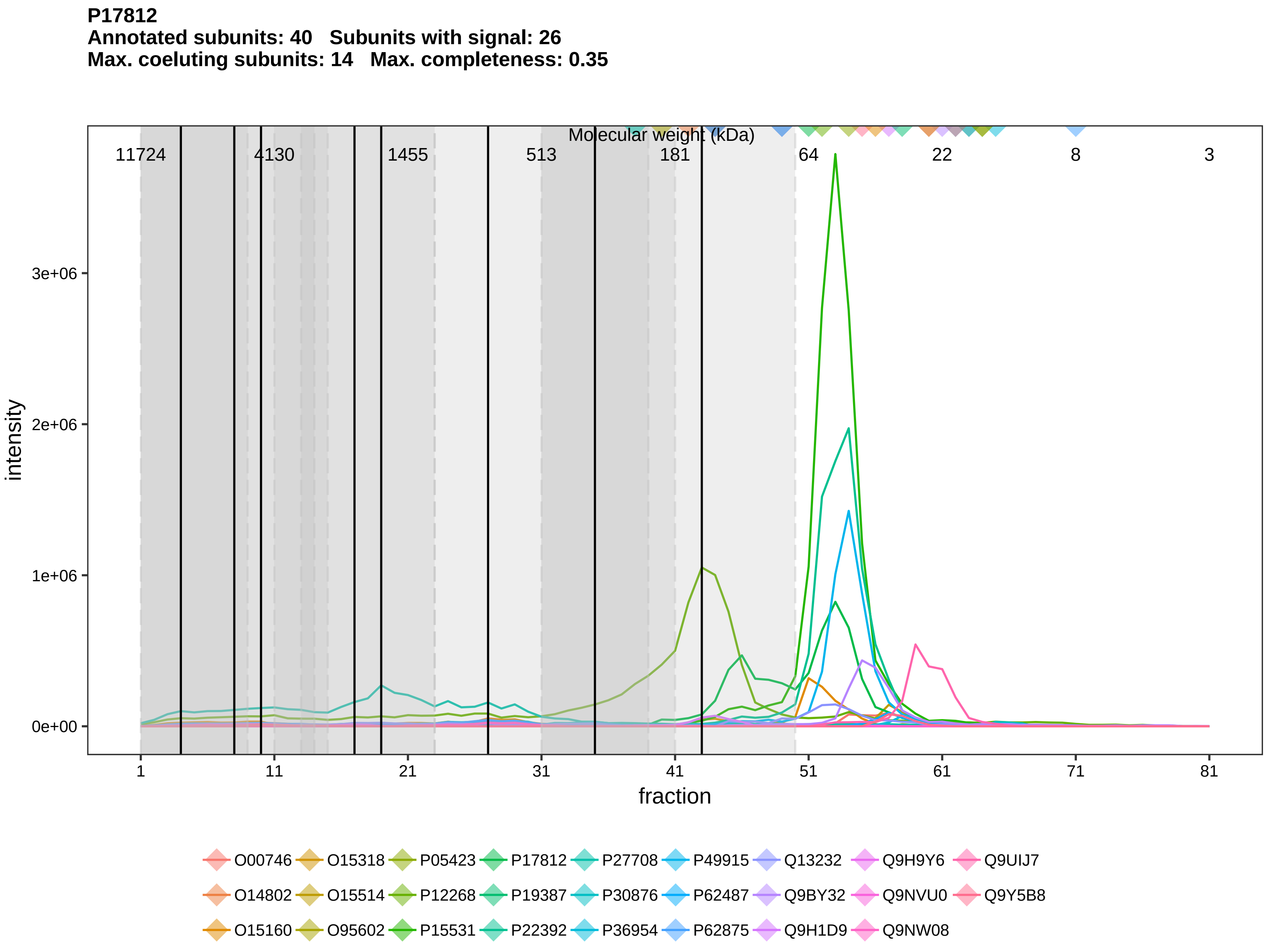
P17812
Annotated subunits: 40   Subunits with signal: 26
Max. coeluting subunits: 14   Max. completeness: 0.35
Molecular weight (kDa)
11724
4130
1455
513
181
64
22
8
3
intensity
3e+06
2e+06
1e+06
0e+00
1
11
21
31
41
51
61
71
81
fraction
O00746
O14802
O15160
O15318
O15514
O95602
P05423
P12268
P15531
P17812
P19387
P22392
P27708
P30876
P36954
P49915
P62487
P62875
Q13232
Q9BY32
Q9H1D9
Q9H9Y6
Q9NVU0
Q9NW08
Q9UIJ7
Q9Y5B8
